# Twelve tips for developing and supporting generic online training for health and medical researchers

**DOI:** 10.15694/mep.2017.000206

**Published:** 2017-11-16

**Authors:** Rashmi Watson, Ali Fardinpour

**Affiliations:** 1 The University of Western Australia

**Keywords:** Training, education, online, curriculum, elearning

## Abstract

This article was migrated. The article was marked as recommended.

Developing online training for health and medical researchers across multiple sites and institutions requires careful consideration to meet the needs of all learners. The Research Education and Training Program (RETP) has attempted to provide a suite of learning modules which are freely accessible to the 21 partner institutions of the Western Australian Health Translation Network (WAHTN). Many lessons have been learnt along the way of this multi-stage process. This paper aims to provide 12 tips in three key areas of online development: writing, online development and maintenance of learning modules. The tips provide a brief summary of these key areas to enable others who may be considering developing and delivering a broad scope of learning modules. This paper is written in the context of modules being delivered for the purpose of continuing professional development and not for high-stakes university courses or similar course requirements.

## Writing the course, Tip 1: Select a Learning Management System (LMS)/Web Host

For an online course to be accessible to potential participants at both home and work sites requires a simple and efficient technology to enable this to happen. A useful task is to complete a scoping exercise into the various Learning Management Systems/Web hosting services to specifically meet the needs of your course requirements as there are many LMSs and each offer varying degrees of abilities and costs. Ideally, the decision should not be limited by cost, available qualified staff or the current system’s requirements and instead on need. However, in reality the choice may be limited by any number of these considerations ( Anderson 2008).

The RETP was part of a larger university system which already provided a Learning Management System. However, the LMS did not suit the needs of the online training we were providing as the RETP modules are not formal, education units of study and hence the requirements were less demanding. A quick scoping exercise to some well-known LMS providers and discussions with colleagues who were using similar providers were beneficial. It is important to see what online development they have already completed for other clients and the level of support they are willing to provide. The big tip here is get everything in writing from the LMS provider as to what exactly is provided and what the cost will be and any additional charges for additional tools known as ‘plugins’. Do not get caught out by not doing your homework first. Our team went with a well-known LMS, and we have received excellent support throughout our journey. However, you have to implement strong pedagogical foundations to facilitate the most desirable use of the provided tools by the LMS ( Govindasamy 2001).

## Tip 2: Project manage course development

The process of online course development does not happen overnight and requires careful planning and project management skills to ensure smooth delivery. If your unit budget allows you to employ a project officer/manager, then it will greatly benefit the final delivery of the courses in a timely and efficient manner. The process of skills into place to ensure that this long process of development from sourcing content experts to the LMS to designing the course to maintaining and evaluating the course must all be planned ahead to ensure smooth delivery. “Project management skills and processes are needed to ensure that rapid eLearning products are delivered successfully within ambitious timelines” (Taran 2006). In the age of IT, proper management and guidance allows for preparedness of up-to-date data for learners leading to the enhancement of improved health for the society ( Emami 2009).

## Tip 3: Identify the curriculum (content)

The ‘curriculum’ or topic development matters as this is what will directly impact the participants as the total learning experiences’ ( Bilbao et al. 2008). Whilst it may sound simplistic to identify topics, it is the first step to successful online course development. The curriculum can be defined as the subject matter, the planned learning experiences, and the intention of learning outcomes and as a cultural reproduction of acceptable knowledge.

Once topics are identified, planning through project management (Tip #3) can be implemented. In our context, we were developing research training modules for health and medical professionals and clinicians including novice researchers, mid-career and to established researchers. The topics need to be selected carefully and mapped out so that topics that were most needed could be developed the earliest. An endless number of online research training topics could be developed, but it is important to have a plan for topic development based on participants’ needs.

## Tip 4: Identify content experts and provide acknowledgement

Sourcing key content experts is critical in ensuring a high-quality learning course can be written, reviewed and revised before online release. When developing an online training course that requires participants to access as much information from the online mode requires that information will be a need to written in such a way that participants can learn and access the necessary information without having a facilitator on hand to support that learning. Content experts can be sourced for a range of roles in the first phase of development including course advisers, writers and course reviewers. To ensure a quality product, quality experts are necessary. According to Govindasamy (2001) not all faculty members and instructors are e-learning content experts. Health professionals such as doctors, nurses and health researchers should also train to learn how to shape their knowledge in a suitable form as an e-learning content.

The RETP is a collaborative network of 21 partners (including five universities and five hospitals) with many suitable course experts who could possibly contribute to the development of courses. However, the reality of the situation is that health and medical clinicians and professionals are busy people working long hours in their usual day jobs and some form of acknowledgement for their time and contribution is necessary to make the process work. This can take place in a number of ways depending on the individual that is contributing. If your budget allows then having a standard payment or honorarium is a way to get people on board willingly. As much as professionals would like to give up time from their usual day-to-day work to support a positive project, in reality, it does not work. When the RETP commenced, it was envisaged that staff would provide voluntary support, but this has not been the case due to the busy nature of today’s world and ability to offer such dedicated time to a task that requires time.

## Tip 5: Write, review and revise the learning and assessment material

Once the initial course structure has been decided by a content expert, written by perhaps a consultant medical writer and then reviewed by identified key experts, additional review and revision is necessary from the core team delivering the course. The tip in ensuring a quality product emerges requires careful review and revision at many points and by many people. Regardless of the level of expertise, individuals will miss aspects that perhaps should or should not be included and errors in writing style.

### Assessment Decisions

An essential part of any learning delivery process is knowing whether the learners are learning. Every course will be different where some will require a basic competency-based assessment or higher level, higher stakes assessment tasks as in university courses. In an online mode, this can be even more difficult in the absence of a facilitator to assess learning in more than one way. In the context that our courses are developed, the assessments are a competency-based quiz on completion of the course that requires participants to pass at 80 percent before being able to successfully pass the module and receive a certificate of completion. The tip is to be clear about what knowledge, skills or attitudes need or must be assessed and then to make decisions on the way this can be assessed. In an online mode, the options are varied and multiple from simplistic short quizzes, multiple choice tests, open-ended tasks, discussion boards to more complex and demanding assessments that may require portfolio submissions or lengthy written assessments. In our context of providing short professional development modules for the purpose of continuous learning of knowledge. A consideration for online course developers is whether the course requires learners to be assessed.

## Tip 6: Utilise trained educational and technical developers

This particular tip should not be underestimated as it impacts directly on the quality of the online product seen by your clients at the other end. Taking complex health content into an online format where in our case there is no contact with a facilitator, requires careful educational design from experts in the area; the first part of the tip. The second component is, having the right technical experts who both understand the LMS/Web source you have selected and the ability to use their technical programming/coding and technical problem-solving skills at all times. Technical issues arise on a regular basis, and it is the technical support staff members that our unit has relied upon on many occasions to navigate through issues that are unknown until participants are actively participating in the courses.

## Tip 7: Transformation (written to digital): Getting the balance right

When working with heavy content such as health and medical research, many want to present excessive amounts of information. The tip here is, set the learning outcomes from the outset by having three to five key areas you would like your learners to know by the time they complete your course to keep to the plan. If you need to provide additional information, then create another module/course that is attached to that course for those that are seeking additional information. Getting the balance right between the amount of content presented, the language of the content presented as professional yet simple enough to understand but not “dumbed down” requires careful writing and transformation. Your participants are there to learn. Use the technical language they need to learn but explain it and use examples to keep the balance right.

## Tip 8: Present material in small chunks and using a range of visual tools

This tip relates to Tip #6. Once you know what information you want and need to tell your online learners, keep the information in small learning ‘chunks’. Chunking is the process of dividing the content into small logical units ( Lynch 2008). Chunking has proved to be a useful principle in content delivery as students’ brain can only process information in small amount at a time ( Weinschenk 2011). Aesthetic design of e-learning courses makes them easier to view and increases the likelihood of use ( Bartuskova and Krejcar 2014) regardless of their ease of use ( Lidwell et al. 2010). Visual tools allow learners to visualise the concepts, map the concepts to visual cues for better remembrance and increase motivation for engagement with the course.

## Tip 9: Explicit and simplified instructions

Once you have your content and learning design in place, it is important to provide clear and explicit instructions for your participants from start to completion. Online learning may be a new experience for many of your learners. Therefore, providing information sheets, instructions at the start of the course, during and at the end of the course, to continue to guide your participants will allow them to feel in control of their learning and supported when you are not there in a face-to-face environment.

## Tip 10: Collect data on learner progress

Knowing that your online learners are satisfied provides a great feeling of accomplishment, however, the learning never ends. This tip links to tip #4 in choosing an online platform that supports the needs and is able to capture data that is important to you. An evaluation of participants’ learning and retention of information is a crucial step in e-learning ( Levac et al. 2015). The data collected should not be limited just to leaner assessment, but also their access to online resources for further analysis ( Levac et al. 2015).

As an example, our team wanted to capture data on specific participant roles and areas of research and which institution they were from. The data provides useful insights into where to target promotion, how to better attract various other users and is useful information for reporting purposes. In our situation, we are required to report to our funding body on a regular basis. Most Learning Management Systems provide tools that allow you to monitor progress of learners whilst they are enrolled.

## Tip 11: Apply changes based on the evidence and lessons learnt

According to the ADDIE (Analyse, Design, Develop, Implement, and Evaluate), constant revision of content improves the quality of the online course ( Branch 2009). “The educational philosophy for this application of ADDIE is that intentional learning should be student centred, innovative, authentic, and inspirational” ( Branch 2009, p. 2). Ongoing review and application of lessons learnt is an essential tip. Ongoing review and application of the changes based on learner feedback and team developers’ insight are critical aspects.

## Tip 12: Timely online support

Technical support for online learners is essential. When participants come across issues, they want and expect assistance quickly. The lack of prompt technical support is one of the reasons for early drop outs in e-learning courses ( Tyler-Smith 2006). Ensure that if participants are having difficulty with technical issues *(e.g. logging in, progressing in the course, generating a certificate)*, help is on hand. Based on your institution, other types of support might be required for the students including educational advising, different types of counselling and help for special needs students ( Anderson 2008).

## Summary

The tips summarise three main stages of the course development and delivery. Before the course delivery, it is important to choose the most suitable learning management system, appropriate project management, identify the curriculum and identify content experts. During the course development, make sure to write, review and revise the learning and assessment material, utilise trained educational & technical developers, transform the written content to the e-learning content, present material in small chunks and using a range of visual tools and explicit and simplified instructions. And finally, after the courses are published, collect data on learner progress, apply changes based on the evidence and lessons learnt and provide a constant timely support.

## Notes On Contributors

Associate Professor Rashmi Watson is the Head of the Research Education and Training Program for the Western Australian Health Translation Network and has worked in various educational contexts for over 20 years.

Dr Ali Fardinpour has an Educational Designer role with the Research Education and Training Program, Western Australian Health Translation Network. He is a research scientist in the field of immersive technologies with interest in health professionals’ education.
