# Supplementary material for: Twelve tips for developing and supporting generic online training for health and medical researchers
Source: MedEdPublish (2016). 2017 Nov 16;6:206. [Version 1] doi: 10.15694/mep.2017.000206 (PMC10885225; doi:10.15694/mep.2017.000206)
Supplement: Peer review history

# Reviewer report: Reviewer response for version 1

**DOI:** 10.21956/mep.19388.r28421

**Published:** 2017-11-19

**Authors:** P Ravi Shankar

**Affiliations:** 1 American International Medical University

This review has been migrated. The reviewer awarded 4 stars out of 5 Online training modules are becoming increasingly important and are being offered across a wide range of settings. At our medical University we do offer our students some online modules designed by institutions in the United States. I had also participated in a few massive open online courses (MOOCs) and enjoyed the experience. Learning management systems are being increasingly used in education. In tip 1 the authors also mention web hosting in the title but do not provide much information about web hosting during the subsequent discussion. Web hosting may be cheaper than investing in a complete learning management system especially for course developers in the developing world. I do agree with the other reviewers that most of the descriptions provided by the authors are generic and could be applicable to various online courses. I would have liked some more description about tip number 7, transformation of material from written to digital. This can be critical as online users access their course material in a variety of settings. Attention spans and concentration are decreasing. Helping the online learners by providing clear and explicit instructions is vital. As mentioned by the authors, different learning management systems have tools to capture data on learner progress. Providing online support in a timely and efficient manner is very important.Many online courses, especially MOOCs employ chat rooms where learners interact in a virtual environment. The facilitators monitor and provide support as and when required. As pointed out by the other reviewer there are errors in the use of language in a few areas. This paper I believe will be of interest to all educators involved with developing online modules or using online modules to supplement on-site teaching-learning.

Reviewer Expertise:

NA

No decision status is available

# Reviewer report: Reviewer response for version 1

**DOI:** 10.21956/mep.19388.r28419

**Published:** 2017-11-16

**Authors:** Ken Masters

**Affiliations:** 1 Sultan Qaboos University

This review has been migrated. The reviewer awarded 3 stars out of 5 A useful paper for those beginning in e-learning. It does, however, have some quite acute shortcomings, as it ignores many issues that can become problematic when developing full-blown online courses, such as selecting and gathering media, checking copyright, etc. (These and other issues cannot be assumed from the paper, for, just as the authors have gone into some detail about using writing experts, so they should also reference media and other experts and their roles). In addition, the online learning envisioned is placed squarely within the confines of an LMS, and quite a bit of online learning has moved beyond the LMS, so, for an audience wider than the novice, it would have been useful if this had also been addressed.Overall, I also felt the effects of some sloppy and rushed writing with irritating errors spoiling the flow of the paper. Some examples:• In Tip 3, you have “planning through project management (Tip #3) can…” I think that “(Tip #3)” should be “(Tip #2)”• Again, in Tip 10, the authors write: “This tip links to tip #4 in choosing an online platform that supports the needs and is able to capture data that is important to you.” Tip #4, however, refers to identifying content experts and providing acknowledgement, so this link is not entirely clear.• While the language is mostly clear, there are many, many minor errors of expression, and the authors should go through their paper carefully correcting these. Just two such small errors: (tense) “The topics need to be selected carefully and mapped out so that topics that were most needed could be developed the earliest”; (number) “shape their knowledge in a suitable form as an e-learning content”. And there are many more, including inappropriate use (or lack) of commas, etc. Finally, the title aims itself at health and medical researchers, but there is very little that is aimed specifically at the health and medical fields. The authors have grounded their work in their own experience in the health and medical fields, but, if one made a simple search and replace of health and medical with engineering or chemistry, the paper would still make complete sense – the topics covered are almost exclusively generic. So, I would have liked to see the paper address issues specifically related to medicine and health (e.g. dealing with issues of protecting patients’ identity when images or videos, or in discussions in the bulletin boards, so crucial to health and medical teaching, are used in online teaching). Or, failing that, remove reference to these fields, and let the paper have wider applicability.

Reviewer Expertise:

NA

No decision status is available

# Reviewer report: Reviewer response for version 1

**DOI:** 10.21956/mep.19388.r28420

**Published:** 2017-11-16

**Authors:** Trevor Gibbs

**Affiliations:** 1 AMEE

This review has been migrated. The reviewer awarded 5 stars out of 5 We are all guilty of starting some sort of project and then seeing it fail because we had not thought it through in a logical manner. This 12 Tips paper attempts to help us in this are and does a good job! I enjoyed reading it, felt that it was very well structured and give personal insights as well as sound academic structures.I felt that the importance of Tip 5 was clearly given, a need to really look at the outcomes expected and revise in relation to the process and need and of course Tip 11 which I feel points to the whole process of evaluation and quality improvement.I believe a must read for those involved in the field

Reviewer Expertise:

NA

No decision status is available
